# *BRAF V600E* mutation and 9p21: *CDKN2A/B* and *MTAP* co-deletions - Markers in the clinical stratification of pediatric gliomas

**DOI:** 10.1186/s12885-018-5120-0

**Published:** 2018-12-17

**Authors:** Laura Frazão, Maria do Carmo Martins, Vasco Moura Nunes, José Pimentel, Claudia Faria, José Miguéns, Amets Sagarribay, Mário Matos, Duarte Salgado, Sofia Nunes, Manuela Mafra, Lúcia Roque

**Affiliations:** 1 Unidade de Investigação em Patobiologia Molecular (UIPM) – IPOFG Cytogenetic Laboratory, Portuguese Cancer Institute, R. Professor Lima Basto, 1099-023 Lisbon, Portugal; 2 Laboratory of Neuropathology, Department of Neurology, Hospital de Santa Maria (CHLN; EPE), Institute of Molecular Medicine, Medicine Faculty of the Lisbon University, Lisbon, Portugal; 3 Neurosurgery Department, Hospital de Santa Maria, Lisbon (CHLN; EPE) Institute of Molecular Medicine, Medicine Faculty of the Lisbon University, Lisbon, Portugal; 4 Neurosurgery Department, Hospital Dona Estefânia, (CHLC; EPE), Lisbon, Portugal; 5 Pediatric Neuro-Oncology Unit, IPOFG, Portuguese Cancer Institute, Lisbon, Portugal; 6 Department of Pathology, IPOFG, Portuguese Cancer Institute, Lisbon, Portugal

**Keywords:** Glial, Pediatric, Tumors, Prognostic, BRAF, 9p21 chromosomal region, *CDKN2A/B*, *MTAP*

## Abstract

**Background:**

Genetic alterations in pediatric primary brain tumors can be used as diagnostic and prognostic markers and are the basis for the development of new target therapies that, ideally, would be associated with lower mortality and morbidity. This study evaluates the incidence and interplay of the presence of *BRAF V600E* mutation and chromosomal 9p21 deletions in a series of 100 pediatric gliomas, aiming to determine the role of these alterations in recurrence and malignant transformation, and to verify if they could be used in the clinical set for stratifying patients for tailored therapies and surveillance.

**Methods:**

Sanger sequencing was used for the assessment of *BRAF* mutations at exon 15 and *Fluorescent* In Situ *Hybridization (FISH)* with BAC: RP11–14192 for the detection of 9p21 alterations. Expression levels of the *CDKN2A* and *MTAP* by real-time PCR were evaluated in cases with 9p21 deletions. Statistical analysis of genetic and clinical data was performed using *Graph Pad Prism 5* and *SPSS Statistics 24* software.

**Results:**

In our cohort it was observed that 7 /78 (8,9%) of the low-grade tumors recurred and 2 (2,6%) showed malignant transformation. *BRAF V600E* mutations were detected in 15 cases. No statistically significant correlations were found between the presence of *BRAF V600E* mutation and patient’s morphologic or clinical features. Deletions at 9p21 abrogating the *CDKN2A/B* and *MTAP* loci were rare in grade I gliomas (12.2%, *p* = 0.0178) but frequent in grade IV gliomas (62.5%, *p* = 0.0087). Moreover it was found that deletions at these loci were correlated with a shorter overall survival (*p* = 0.011) and a shorter progression-free survival (*p* = 0.016).

**Conclusions:**

It was demonstrated that in these tumors *BRAF V600E* mutated and that *CDKN2A/B MTAP* co-deletions may be used for stratifying patients for a stricter surveillance. The Investigating and defining if glial tumors with *CDKN2A/B* and *MTAP* homozygous loss may be vulnerable to new forms of therapy, namely those affecting the methionine salvage pathway, was proven to be of importance.

**Electronic supplementary material:**

The online version of this article (10.1186/s12885-018-5120-0) contains supplementary material, which is available to authorized users.

## Background

Tumors of the Central Nervous System (CNS) account for 20–25% of all pediatric cancer diagnoses in the developed world, being the most prevalent group of cancers in children, after leukemia. Although uncommon, pediatric brain tumors represent the leading cause of cancer-related mortality in children and adolescents aged 20 years and under; and the third leading cause of cancer-related death in young adults aged 20 to 39 years. In addition to their usual high mortality, CNS tumors are associated with extensive morbidities such as pituitary dysfunction, growth hormone deficiency, epilepsy, vision loss, impaired motor skills, memory dysfunction, attention and behavioral disorders and reduced intelligence quotient (IQ). So, about 90% of survivors are left with long term cognitive and psycho-social deficits [1].

Glial and neuro-glial derived tumors are the most frequent CNS tumors in pediatric population [1, 2]. They form an heterogeneous group of neoplasia which are categorized by the World Health Organization (WHO) [2, 3] into various entities based on their cellular, genetic and clinical characteristics.

In what concerns their clinical features, gliomas are clustered in two major groups: low-grade gliomas (LGG) and high-grade gliomas (HGG). LGG comprise: i) the non-diffuse/non-infiltrative pilocytic astrocytoma (PA) categorized as WHO grade I tumor due to its predominantly favorable outcome, which, depending on their localization, can be cured by surgery alone: ii) the diffuse/ infiltrative gliomas which are associated with a less favorable clinical outcome, namely, recurrence after initial resection and a higher probability to malignant transformation. The most frequent histological entities ascertained to the former group are: the diffuse astrocytoma grade II, gangliogliomas, the angiocentric gliomas and pleomorphic xanthoastrocytomas.

Conversely, HGG comprise other histological entities that invariably recur and show progressive disease after the initial surgical resection. In the HGG are included all WHO grade III and IV neoplasms and the most frequent histologic categories are: anaplastic astrocytoma, anaplastic pleomorphic xanthoastrocytoma and glioblastoma (GBM).

In pediatric low-grade gliomas, the analysis of the molecular and genetic mechanisms underlying gliomagenesis allowed for an understanding of *MAPK/ERK* pathway activation as fundamental for their development. This occurs at high frequency by activation of the *BRAF* oncogene; and in lower frequencies: by *MYB* and *MYBL1* amplifications and rearrangements, and *FGFR1* rearrangements and mutations [4–10].

Two different mechanisms may lead to *BRAF activation* in pediatric brain gliomas: chromosomal rearrangements and point mutations. The most common *BRAF* rearrangement is the one resulting in *BRAF/KIAA1549* fusion protein in which the *N-terminus* of the protein encoded by *KIAA1549* gene is fused with the *C-terminus* of the protein encoded by *BRAF* gene, preserving the *BRAF* kinase domain [10, 11]. *BRAF* activating rearrangements were reported to be present in 70% of the pilocytic astrocytomas, in 15% of other low-grade gliomas, and have only been punctually observed in high-grade gliomas [9]. Studies performed by Hawkins et al. (2011) [12], Horbinski et al. (2010) [13], and Jones et al. (2008) [14], showed that *BRAF* rearrangements were an independent favorable prognostic factor in both supra-tentorial and posterior fossa low-grade gliomas.

The vast majority (> 90%) of *BRAF* mutations in pediatric gliomas are *BRAF V600E* mutations, a somatic mutation causing the substitution of the amino acid valine by glutamic acid at residue 600 of exon 15. *BRAF V600E* mutations have been described in a wide variety of lesions: 80% of pleomorphic xanthoastrocytomas 33% of the gangliogliomas, 23% of the diffuse astrocytomas, 10% of the glioblastomas being more frequent in tumors located in the cerebral cortex [15]. Only rarely *BRAF V600E* mutation occurs in conjunction with a *BRAF/KIAA1549* rearrangement in the same tumor [4].

At variance with BRAF rearrangements, the role of *BRAF V600E* mutation in the glioma’s evolution and patient’s follow-up is far from being fully understood and some contradictory results are found in literature. Accordingly, while Horbinski et al. (2012) [13] showed that in their cohort of pediatric low-grade gliomas, *BRAF V600E* mutationended to a worse progression-free survival when compared to wild-type tumors, Mistry et al. (2015) [16] showed that this mutation was associated with a prolonged latency to malignant transformation and, consequently, with a better overall survival when compared to wild-type pediatric low-grade gliomas.

Moreover, Korshunov, et al. (2015) [17] described a subgroup of glioblastomas, exclusive to the pediatric population, that was characterized by the *BRAF V600E* mutation and *CDKN2A* deletion. Although these tumors had a better overall survival, they still had a high recurrence rate (67%).

The *CDKN2A* gene is mapped at the chromosome 9p21 region and encodes the *p16*^INK4A^ and p14^ARF^ proteins. *p16*^INK4A^ protein has a key role as negative regulator of the proliferation of normal cells, controlling the progression through *G1* into the *S phase* of the cell cycle. According to Raabe et al. (2011) [18], the worst outcomes associated with *CDKN2A* gene deletion could reflect a failure to induce senescence or an escape from the induced tumor senescence in *BRAF* driven *CDKN2A*^*−*^ tumors.

In order to further understand the interplay between *BRAF V600E* mutation and the chromosomal region abrogating the *CDKN2A* gene: 9p21, a cohort of 100 pediatric gliomas was retrospectively analyzed.

## Materials and methods

### Human tumor samples

Analysis was performed in paraffin embedded material or fresh tumor samples of patients with pediatric gliomas that were referred to the Portuguese Institute of Oncology (IPOFG-Lisbon, Portugal) from 1992 to 2015 following Patient and Institutional Ethical Board Committee approval.

**Fig. 1:**
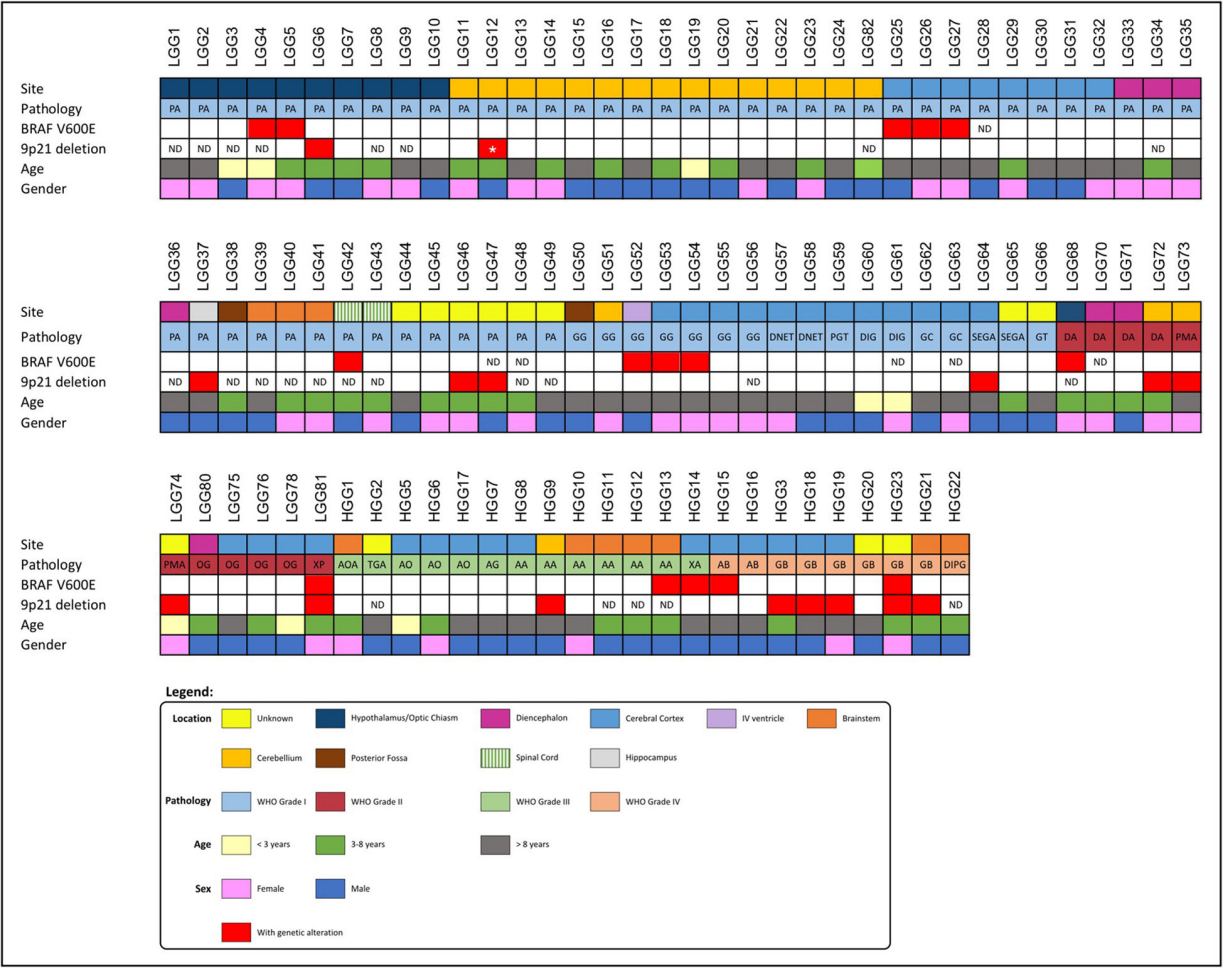
Graphical representation of the clinical data and of the genetic alterations observed in the cohort of pediatric gliomas. Abreviations: AB (Grade IV Astroblastoma), AO (Anaplastic Oligodendroglioma), AOA (Anaplastic Oligoastrocytoma), DA (Diffuse Astrocytoma), DIG (Desmoplastic Infantile Ganglioglioma), DIPG (Diffuse Intrinsic Pontine Glioma); DNET (Dysembryoplastic Neuroepithelial Tumor), GB (Glioblastoma), GC (Gangliocytoma), GG (Ganglioglioma), GT (Glioneuronal Tumor) OG (Oligodendroglioma), PMA (Pilomyxoid Astrocytoma), PA (Pilocytic Astrocytoma), PGT (Papillary Glioneuronal Tumor), SEGA (Subependymal Giant Cell Astrocytoma), TGA (Anaplastic Glioneuronal Tumor), XA (Xanthoastrocytoma), XP (Pleomorphic Xanthoastrocytoma), AA (Anaplastic Astrocytoma), ND (Not Done). Red squares represent the presence of the genetic alteration

In total, 100 pediatric gliomas (PG) were selected for genetic analysis: 67 grade I gliomas, 11 grade II gliomas, 13 grade III gliomas, and 9 grade IV gliomas. Since the aim of this study was to investigate the influence of *BRAF V600E* mutation and 9p21 gene loss, grade I and II lesions were assembled in one group, referred as Low-Grade Gliomas (LGG) and grade III and IV in another group, referred as High-Grade Gliomas (HGG). Histological classification was performed and revised according to WHO (2007) criteria. The patients’ clinical characteristics (tumor’s location, histologic classification, WHO grade, patient’s age-ranges and gender) and outcomes are depicted in Fig. 1 and Additional file 1: Table S1. In grade I gliomas (referred in Additional file 1: Table S1 as LGG1 to LGG66, and LGG82): 32 cases were females and 35 were males, the mean age in this group was of: 9.25 years (range from 5 months to 17 years); In grade II gliomas (referred as LGG68; LGG70 to 76; LGG78, 80 and 81 in Additional file 1: Table S1): 6 cases were females, 5 males, the mean age in this group was: 6.02 years (range from 1 year to 17 years). As to grade III gliomas (referred in Additional file 1: Table S1 1 as HGG1–2; HGG5 to 15): 3 were females, 10 were males and the mean age of this group was: 9.36 years (range from 9 months to 16 years). In grade IV glioma group of this study (referred in Additional file 1: Table S1 as HGG3; HGG16 to 23): 2 patients were females and 7 males, the mean age in this group was 10.44 years (range from 5 years to 15 years).

In accordance with WHO 2007 criteria, patients were considered to have a recurrent tumor when exhibiting a glioma of the same histologic grade after at least 1 year of remission. They were considered to have a de novo tumor when exhibiting a glioma of the same grade, with a different histological type and at a different localization. Patients were only considered as having a transformation of LGG to HGG if they exhibited one of the following features: i) consecutive histologic diagnosis of LGG and HGG as per the WHO 2007 criteria, ii) histological diagnosis LGG follow by at least 1 year of stable disease, followed by clinical and radiological progression to HGG.

### Fluorescent in situ hybridization (FISH)

FISH technique was used to identify heterozygous and homozygous deletions of the 9p21 chromosomal region. It was performed in interphase nuclei with DNA probes indirectly labeled. A locus-specific BAC probe (RP11–14912) directed to the chromosomal location 9p21.3 within chromosome 9–21,909,260 and 22,010,414 (Ensembl *Homo sapiens* version 86.38 (GRCh38.p7) - was used to identify the 9p21 chromosomal region. An enumeration probe for chromosome 9 (pMR9A) was also used. The 9p21 chromosomal region probe was labeled with digoxigenin and the chromosome 9 probe with biotin using the BioPrime^R^ DNA Labeling System (Invitrogen™,by Thermo Fisher Scientific, USA). These labels were then detected with specific antibodies: anti-digoxigenin-fluorescein (Roche Diagnostics, Germany) and streptavidin combined with cyanine 3 (Cy3) fluorophore (Jackson ImmunoResearch Laboratories, USA), respectively. FISH signal evaluation was performed in an AxioImager fluorescence microscope linked to aCytoVision® software (Applied Imaging, UK). Analysis was performed by counting at least 100 cells per slide with intact, non-overlapping nuclei taken from different, randomly chosen fields of view. Deletion was defined as more than 40% containing 9p21 probe: cep9 probe ≤0.5.

### Sequencing using sanger methodology

In the context of this work, sequencing using Sanger methodology was used to identify a single point mutation (V600E) in the exon 15 of the *BRAF* gene. DNA for gene analysis was extracted by different procedures according to the material used (fresh material or paraffin embedded material). Target gene amplification was achieved through polymerase chain reaction (PCR), using the forward primer ^5’^TCATAATGCTTGCTCTGATAGGA^3’^ and the reverse primer ^5’^CCGGTTTTTAAATTAGTCACCT^3’^ at an annealing temperature of 58–59.5 °C. To sequence samples, an automatic sequencer, ABI PrismTM 3130 Genetic Analyser (Applied Biosystems), was used. Electrophoretograms were compared with the reference DNA sequence of the studied gene to identify the presence or absence of mutations. Reference DNA sequences were obtained from Ensembl data base, available at: http://www.ensembl.org/index.html.

### Expression studies using quantitative real-time RT-PCR

In cases with 9p21 deletions (detected by FISH) expression analysis was performed by real-time qPCR, using commercial TaqMan probes for the *CDKN2A* (Hs00233365_m1) and *MTAP* (Hs00559618_m1) genes (ThermoFisher Scientific, USA). Analysis was performed using QuantStudio5-Applied Biosystems Real-Time PCR System. *ABL1* probes as described by Beillard E et al. [19] were used as endogenous control for gene expression assays. RNA from 3 sample cases (CN1, CN2, and CN3), defined as non-neoplastic brain lesions by pathology, were used as calibrator controls. We were not able to extract RNA with sufficient quality and purity, as analysed by agarose gel electrophoresis and Nanodrop spectrophotometry (NanoDrop Technologies,USA), from all cases with 9p21 loss of this cohort. Total RNA extraction and single-strand cDNA synthesis could only be performed for 3 LGG presenting 9p21 heterozygous loss: LGG64, LGG73 and LGG74 and for 3 HGG (grade IV) with 9p21 homozygous loss: HGG3, HGG18 and HGG23. Total RNA was extracted from 30 mg of the tumor specimens using the RNeasy® Mini Kit (Qiagen) and c-DNA synthesis was performed using the superscript II kit (Invitrogen, ThermoFisher Scientific). Gene expression was quantified on QuantStudio5 (Applied Biosystems, ThermoFisher Scientific, USA). Real-Time PCR System was performed as per manufactures protocol. *CDKN2A* and *MTAP* expression were normalized with to reference gene ABL1 and the fold change relative to CN1, CN2, CN3 samples was calculated.

### Statistical analysis

Statistical analyses were carried out using *GraphPad Prism 5* software (San Diego, USA). The two-tailed Fisher’s exact test was used to determine the correlations between the presence or absence of genetic alterations and patients’ gender, tumors’ WHO grade, histology and location. The Mann-Whitney test was used to study the presence or absence of genetic alterations with patient’s age. Survival curves were compared using the Log-Rank (Mantel-Cox) test using the *IBM SPSS Statistics 24* Software. Differences were considered statistically significant at *p*-value < 0.05.

## Results

### *BRAF V600E* mutation

In total, 94 pediatric gliomas were analyzed for the *BRAF V600E* mutation and 15 were found to harbor this mutation (Fig. 1 and Additional file 1: Table S1): 9/62 (14,5%) grade I gliomas (6 pilocytic astrocytomas (PA) and 3 gangliogliomas (GG)); 2/11 (18,2%) grade II gliomas (1 diffuse astrocytoma (DA) and 1 pleomorphic xanthoastrocytoma (XP) classified as a grade II lesion); 2/13 (15,4%) grade III gliomas (1 anaplastic astrocytoma (AA) and 1 anaplastic XP); and 2/9 (22,2%) grade IV gliomas (one astroblastoma and one glioblastoma (GBM)).

The presence of *BRAFV600E* mutation did not show any correlation with tumor’s WHO grade, histologic subtype, patients’ age (*p =* 0.9299) and patients’ gender (*p* = 0.1539). This mutation was not found in tumors located in the cerebellum (*p* = 0.0355).

**Fig. 2:**
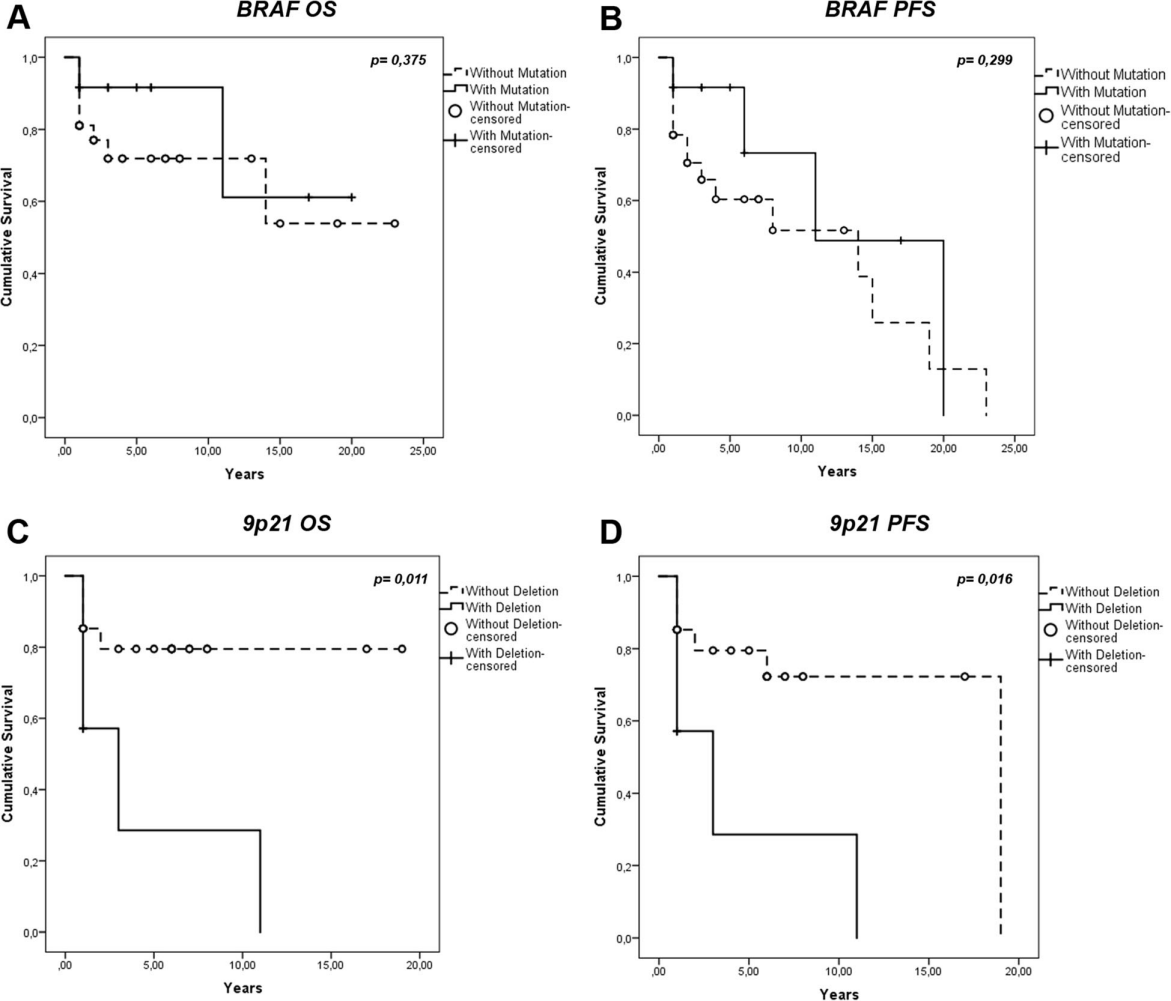
Correlation between *BRAF* and 9p21 alterations and the clinical outcome - Kaplan-Meier curves. **a:** Overall Survival (OS) curves of *BRAF V600E* – positive tumors *v.s*. *BRAFwt* tumors, *p* = 0.375; **b:** Progression-Free Survival (PFS) curves of *BRAF V600E* – positive tumors *v.s. BRAFwt tumors, p =* 0.299; **c:** OS curves of the 9p21 *chromosomal region* deleted tumors *v.s* those without 9p21 deletion, *p* = 0.011; **(d)** PFS curves of the 9p21 *chromosomal region* deleted tumors *v.s* those without 9p21 deletion, *p* = 0.016

Regarding the correlation of *BRAF V600E* mutation with the overall survival rate of the patients, although no statistically significant correlation could be observed (*p* = 0,375), a trend toward a better overall survival (OS) was detected (Fig. 2). No statistically significant correlation (*p* = 0,299) was observed when analyzing progression free survival (PFS) differences between *BRAF* wild type (*BRAFwt*) and *BRAF V600E* mutated (*BRAFmut*) tumors (Fig. 2).

### 9p21 chromosomal region deletion

For the 9p21 chromosomal region, 76 gliomas were analyzed, and its deletion was identified in 16 cases, as depicted in Fig. 1 and Additional file 1: Table S1. 9p21 chromosomal region deletions were observed in 10/59 (16,9%) LGG (cases LGG: 6, 12, 37, 46, 47, 64, 72 to 74 and 81) in which all deletions were heterozygous. 6/17 (35,3%) HGG (HGG: 3, 9, 18, 19, 21 and 23) presented 9p21 deletions, four of which had a homozygous 9p21 chromosomal region deletion.

Statistical analysis demonstrated that 9p21 chromosomal region deletion did not correlate with tumor location, patients’ age (*p* = 0.6093) and patients’ gender (*p* = 0.7831), but it did correlate with gliomas’ grade. The 9p21 chromosomal region deletion was rare in grade I gliomas (12.2%, *p* = 0.0178) but frequent in grade IV gliomas (62.5%, *p* = 0.0087). Moreover, this deletion also correlated with the histology categorization of tumors, a *p = 0 .0012* was found between 9p21 loss and glioblastoma histology.

Furthermore, as depicted in Fig. 2 the 9p21 chromosomal region deletion was correlated with a worse overall survival rate (*p* = 0.011). The PFS values of 9p21 loss group were also significantly (*p* = 0.016) worse than those of the group with no deletion at 9p21 as observed in Fig. 2.

### *BRAF V600E* and 9p21 deletion, in recurrence, progression and development of secondary HGG

Of the 100 gliomas evaluated in our series during a 23-year period it was observed that in the 78 LGG group, 7 recurred (8,9%) (LGG 2, 3, 34, 36, 40, 54, 68) and 2 (2,6%) showed progression to HGG (LGG81 and LGG82) (Table 1). It must be noted that the development of gliomas in the patient identified as LGG68 was very interesting. This patient (ruled out as having Neurofibromatosis type 1) was diagnosed at the age of 4 with a diffuse astrocytoma of the optical nerve, which was positive for the *BRAF V600E* mutation and presented a recurrence, 3 years later, that also was positive for *BRAF V600E*. However, at the age of 5 (two years before recurrence) he also had a pilocytic astrocytoma, which developed at the cerebral cortex that did not present *BRAF V600E* mutation. These findings suggest that the cortical and the optical nerve’s LGG of the patient had different cells of origin. This tumor was therefore considered as de novo glioma.

**Table 1:** Summary of the genetic findings in glioma cases that recurred, progressed to HGG or presented a de novo tumor

	Patient N° / Condition	Time of Biopsy / Surgery	Age (years)	Location	Diagnosis	*BRAFV600E* Mutation	9p21 Chromosomal Region Deletion (9p21 region//centromere of chromosome 9)	Follow-up
1	LGG3	1992	< 3	Hypothalamus/Optic Chiasm	Pilocytic Astrocytoma	Negative	Negative	Alive, 2015
	LGG10	2009	> 8	Hypothalamus/Optic Chiasm	Pilocytic Astrocytoma	Negative	Negative	
2	LGG68	1995	3–8	Hypothalamus/Optic Chiasm	Diffuse Astrocytoma	*Positive*	ND	Alive, 2015
	LGG29	1996	3–8	Cerebral Cortex	Pilocytic Astrocytoma	Negative	Negative	
	LGG5	1999	3–8	Hypothalamus/Optic Chiasm	Pilocytic Astrocytoma	*Positive*	Negative	
3	LGG40	2000	3–8	Brainstem	Pilocytic Astrocytoma	Negative	ND	Alive, 2015
	LGG41	2002	3–8	Brainstem	Pilocytic Astrocytoma	Negative	ND	
4	LGG34	2011	3–8	Diencephalon	Pilocytic Astrocytoma	Negative	ND	Alive, 2015
	LGG48	2013	3–8	Unknown	Pilocytic Astrocytoma	ND	ND	
5	LGG82	1996	3–8	Cerebellum	Pilocytic Astrocytoma	Negative	ND	Death, 2010
	HGG9	2009	> 8	Cerebellum	Anaplastic Astrocytoma	Negative	*Positive*	
6	LGG54	2009	3–8	Cerebral Cortex	Ganglioglioma	*Positive*	Negative	Alive, 2015
	LGG53	2014	> 8	Cerebral Cortex	Ganglioglioma	*Positive*	Negative	
7	LGG2	2013	> 8	Hypothalamus / Optic Chiasm	Pilocytic Astrocytoma	Negative	ND	Alive, 2015
	LGG9	2015	> 8	Hypothalamus / Optic Chiasm	Pilocytic Astrocytoma	Negative	ND	
8	LGG36	2014	> 8	Dienchephalon	Pilocytic Astrocytoma	Negative	ND	Alive, 2015
	LGG37	2015	> 8	Hippocampus	Pilocytic Astrocytoma	Negative	*Positive*	
9	LGG81	2011	3–8	Cerebral Cortex	Pleomorphic Xanthoastrocytoma	*Positive*	*Positive*	Death, 2014
	HGG23	2013	> 8	Cerebral Cortex	Glioblastoma	*Positive*	*Positive*	

*LGG* Low-grade Glioma,*HGG* High-grade glioma, *ND* Not Done

In grade III HGG group, five patients showed progressive disease and died (cases HGG1, 8, 9, 10 and 11), 4 patients were alive (HGG5, 6, 12 and 14) and in 3 cases follow-up was lost.

As for grade IV HGG group (*n* = 9) three patients died (HGG 3, 18, 23) of progressive disease. HGG18 was considered a secondary HGG. This patient had been diagnosed in 1998 at age of 4 with acute lymphoblastic leukemia type B (ALL-B), received a medullar transplant and was treated with radiotherapy. He was considered at remission between 2003 and 2009 but, in 2009 at the age of 15, he was diagnosed as having a glioblastoma. Of the other grade IV gliomas of our series, two (HGG15 and 16) were still alive in 2015. Both tumors were classified as astroblastomas. One, HGG15 had a *BRAF V600E* mutation. However, none of them presented a deletion of 9p21 region. Four cases (HGG19, 20, 21, 22) were lost for follow-up.

### *CDKN2A* and *MTAP* expression studies by quantitative real-time RT-PCR

The expression levels for the *CDKN2A* and *MTAP* genes in both the LGG and HGG are depicted in Additional file 2: Figure S1 and in Additional file 3. In the LGG cases expression levels for the *CDKN2A* gene mRNA levels were normal or elevated. A similar situation was observed for the *MTAP* gene for cases LGG64 and LGG73. However, in LGG74, identified as pilomyxoid astrocytoma BRAFwt, *MTAP* gene levels were slightly down-regulated. In all HGG, expression levels for both genes were significantly down-regulated.

## Discussion

Incidence and interplay of the presence of *BRAF V600E* mutation and 9p21 region deletion was evaluated in a series of 78 LGG and 22 HGG aiming to determine the role of these two alterations in recurrence and gliomas’ malignant transformation. There was also interest in verifying if they could be used in a clinical set for stratifying patients for existing target therapies and a tailored strict surveillance.

As to the incidence rate of *BRAF V600E* mutation results corroborated previous data [4, 15, 17], showing that this mutation occurred in all spectra of glioma types, although being more frequent in gangliogliomas and diffuse astrocytomas. Accordingly, we identified *BRAF V600E* mutation in 43% of the gangliogliomas, in 33% of the diffuse astrocytomas in 13% of the pilocytic astrocytomas, but also in one anaplastic astrocytoma and one glioblastoma.

In our population we observed a recurrence incidence of 8.9% and a 2,6% incidence of pediatric LGG, which undergo malignant transformation into HGG.

Statistical evaluation revealed no correlation between the presence of *BRAF V600E* and recurrence or progression in our series.

Mistry, et al. (2015) [16] by analysis of a cohort of 886 patients reported a similar incidence rate of pediatric LGG transformation to sHGG - 2,9%, but at variance with our data they showed *BRAF V600E* mutation to be associated with prolonged latency periods in pediatric low-grade gliomas. Our data suggests that tumor progression is independent of the presence of *BRAF V600E* mutation since progression free survival rates were very similar between the two groups: *BRAFwt* and *BRAFmut*.

In our series 9p21 deletion was rare in grade I (12.2%, *p* = 0.0178) but frequent in grade IV gliomas (62.5%, *p* = 0.0087). Moreover, this deletion also correlated with the histologic categorization of tumors, a *p = 0.0012* was found between 9p21 deletion and glioblastoma histology.

In accordance with previous studies performed by Horbinski et al. (2012*)* [13], Mistry et al. (2015) [16] and Roy et al. (2016) [20] results led to the conclusion that homozygous or heterozygous deletions of the 9p21 chromosomal region were associated with a shorter overall survival and with a worse progression-free survival.

Interestingly, the two pediatric LGG (LGG81 and 82) that underwent malignant transformation into HGG (HGG 23 and 9 respectively), both presented a 9p21 region deletion. One, LGG81, also had a *BRAF V600E* mutation ab initio and the observed latency period until the development of the HGG was two years. LGG82 did not present ab initio a *BRAF V600E* mutation and had a much longer latency period until the development of the HGG (13 years). Although the number of cases is recognizably small, these findings suggest that in LGG81, a cooperative interplay might have occurred between *BRAF* mutation and 9p21 homozygous loss, which enhanced malignant transformation.

Several tumor suppressor genes (TSG) are known to be located at 9p21, namely, the Cyclin Dependent Kinase Inhibitor’s: *CDKN2A* and *B*; the Kelch Like Family Member 9 (*KLHL9*) and the Metylthioadenosine Phosphorilase (*MTAP*). The role of all these TSG in gliomas has been under investigation. Huilard et al. (2012) [21] by performing functional studies in neural progenitor cell and mouse models evidenced that homozygous *CDKN2A/INK4a-Arf* deletion in *BRAF V600E* expressing cells was sufficient for the formation of tumors with histologic features similar to the malignant astrocytomas in humans [21]. Mistry et al. (2015) [16] in an array of comparative genomic hybridization analysis of 886 pediatric gliomas alsodefined that *CDKN2A* deletions had a central role in low-grade glioma malignant transformation. On the other hand, Roy et al. (2016) [20] in a cohort of 379 adult LGG, conversely observed in their genomic analysis that *CDKN2A* inactivation was not associated with progression. Indeed, by correlating gene expression with zygosity status, they found that *CDKN2A* expression levels were normal or even elevated when associated at heterozygous gene loss, and proposed rather that *KLHL9* and *MTAP* would concurrently and in a context-dependent manner promote tumor aggressiveness.

In this study BAC-RP11–14912 was used for FISH analysis. This BAC has a 101,115 bps genomic insert that encompasses not only *CDKN2A/B,* but also the *MTAP-009* gene segment, which encodes a 112 amino acid *MTAP* protein. The detection of losses of this DNA segment in our cases was indicative that both genes could be involved in the malignant transformation of pediatric LGG. By performing expression analysis by qPCR for both the *CDKN2A* and *MTAP* genes in cases referred in our series as: LGG 64; 73; 74 and HGG 3; 18; 23, it was evidenced that in the LGG characterized by heterozygous gene losses there was still a “normal” expression of both genes, but in HGG cases, the homozygous loss was associated with a significant down-regulation of both *CDKN2A* and *MTAP* genes. The observation that *MTAP* is also involved in pediatric gliomas is of foremost importance since in very recent surveys [22, 23] it was revealed that *MTAP* deleted cancers were rendered therapeutically vulnerable at the methionine salvage pathway. Accordingly, to Mavrakis et al. (2016) [22] and Marjon K et al. (2016) [23] deletions at 9p abrogating the *MTAP* gene lead to the accumulation of its substrate 5′ methilthioadenosine (MTA) and a metabolic “rewiring” of the tumor cells, which rendered them specifically sensible to drugs that inhibit the methyltransferase axis composed by: Methionine Adenosyltransferase II alpha (*MAT2A*), Protein Arginine Methyltransferase 5 (*PMRT5*) and Rio domain containing protein 1 (*RIOK1*).

As aforementioned, HGG18 case of our cohort (Table 1) was diagnosed as a secondary glioblastoma following medullar transplant and radiotherapy of an ALL-B. Reports on the genetic analysis of secondary CNS tumors in children and their previous ALL are exceedingly rare, and the driver versus passenger mutations that underlie leukemia metastasis and the development of secondary tumors are still to be defined [24–26]. HGG18 was negative for the presence of *BRAF V600E* mutation but positive for 9p21 deletion (Table 1). No molecular evaluation for these two genetic alterations could be retrospectively performed in the leukemia cells, but the karyotype observed in bone marrow blasts at relapse revealed a hiperdiploid karyotype, with 54 chromosomes and a gain of two copies of chromosome 9. Although, it could not be ruled out experimentally that in this case a specific *CDKN2A/B* and *MTAP* deletion or epigenetic silence of these TSGs were not present ab initio and at relapse in the leukemia cells of the patient, it is possible to consider that in the CNS microenvironment, but not in the medullar microenvironment, *CDKN2A/B/MTAP* loss and down-regulation contributed to expansion of the patients neoplastic clone, therefore reinforcing the view that chromosomal deletions encompassing these genes are important for glioma progression.

In summary our data allowed us:

i) To verify that the role of *BRAF V600E* mutation is dependent of other co-occurring genetic alterations, specifically 9p21 deletions that abrogate both the *CDKN2A/B* and the *MTAP* tumor suppressor genes;
ii) To demonstrate that 9p21 deletions correlated significantly with poor prognosis indicators and its detection could be used to stratify patients for a stricter surveillance;
iii) To underline the relevance of investigating and defining if glial tumors with *CDKN2A/B* and *MTAP* loss may be vulnerable to new forms of therapy, namely those affecting the methionine salvage pathway.

## Additional file

Additional file 1: **Table S1.** Summary of the Clinical and Genetic Data of Glioma Cases. (XLSX 28 kb)

Additional file 2: **Figure S1.** Graphical representation of the expression of the *CDKN2A* and *MTAP* genes as determined by qPCR in pediatric Low-Grade Gliomas (LGG: 64, 73, 74) and High-Grade Gliomas (HGG: 3, 18, 23 of our series). CN1–3 – non-neoplastic brain lesions used as calibrator controls. (JPG 360 kb)

Additional file 3: Excel files of TaqMan experiments for the *CDKN2A* and *MTAP* genes. (XLSX 127 kb)

## Abbreviations

ABL1: ABL Proto-Oncogene 1
BAC: Bacterial Artificial Chromosome
BRAF: B-RAF Proto-Oncogene, Serine/Threonine Kinase
*CDKN2A*: Cyclin Dependent Kinase Inhibitor 2A
CDKN2B: Cyclin Dependent Kinase Inhibitor 2B
ERK: Extra Signal-Regulated Kinases
KLHL9: Kelch Like Family Member 9
MAPK: Mitogen Activated Protein Kinase
*MTAP*: Methylthioadenosine Phosphorilase
MYB: MYB Proto-Oncogene, Transcription Factor
MYBL1: MYB Proto-Oncogene Like 1
