# Supplementary figures and images for: BRAF V600E mutation and 9p21: CDKN2A/B and MTAP co-deletions - Markers in the clinical stratification of pediatric gliomas
Source: BMC Cancer. 2018 Dec 17;18:1259. doi: 10.1186/s12885-018-5120-0 (PMC6296141; doi:10.1186/s12885-018-5120-0)

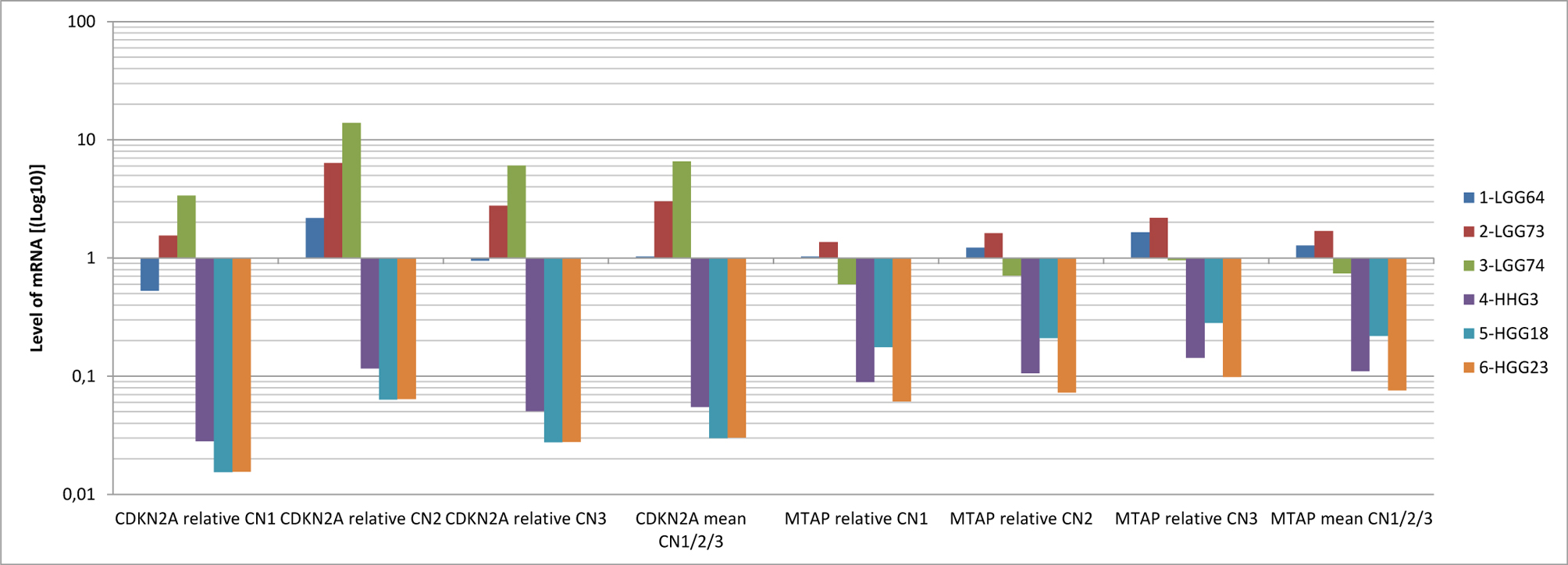

Supplement: Supplementary file 2 — Figure S1. Graphical representation of the expression of the CDKN2A and MTAP genes as determined by qPCR in pediatric Low-Grade Gliomas (LGG: 64, 73, 74) and High-Grade Gliomas (HGG: 3, 18, 23 of our series). CN1–3 – non-neoplastic brain lesions used as calibrator controls. (JPG 360 kb) [file 12885_2018_5120_MOESM2_ESM.jpg]
